# Antibody–drug conjugate: a newly developed biological missile for tumor treatment

**DOI:** 10.3389/fonc.2025.1688057

**Published:** 2025-10-29

**Authors:** Zhuoran Tang, Yanchun Xie, Yanping Zeng

**Affiliations:** ^1^ Mudi Meng Honors College, China Pharmaceutical University, Nanjing, China; ^2^ Department of Neurology, Renmin Hospital of Wuhan University, Wuhan, China

**Keywords:** antibody drug conjugate, monoclonal antibody, linker, cytotoxic payload, resistance, toxicity, immune checkpoint inhibitor

## Abstract

Antibody–drug conjugates (ADCs) represent one of the most advanced drug configurations under current research, primarily composed of a monoclonal antibody (mAb), a highly potent cytotoxic payload, and a linker that connects the drug to the antibody. The mAb serves mainly as a targeting moiety, guiding the conjugate to specific cells. The cytotoxic payload is responsible for the anticancer activity, whereas the linker ensures stable attachment between the antibody and the payload during circulation. The core advantage of ADCs lies in their ability to leverage the specificity of antibodies to deliver highly potent cytotoxic agents precisely to tumor cells, thereby significantly improving the therapeutic index. However, they also face challenges such as systemic toxicity, drug resistance, tumor heterogeneity, and complex manufacturing processes. Currently, extensive research is focused on technological innovations, the development of novel ADCs, and the optimization of clinical combination therapies. This article provides a comprehensive review of the structure and mechanism of action of ADCs, their developmental history, current challenges, emerging novel agents, and combination strategies with immune checkpoint inhibitors (ICIs).

## Introduction

Every year, cancer causes more than 8.2 million deaths worldwide, making it the second leading cause of death globally and a major public health threat (1, 2). For a long period of time, conventional treatments such as radiotherapy and chemotherapy have served as the basis for managing a wide range of cancers (3, 4). However, these approaches lack sufficient specificity and have a narrow therapeutic index, often leading to severe side effects due to non-specific drug exposure in healthy tissues (5). Thus, there is a critical need to develop novel cancer therapeutics with improved targeting capabilities.

**Figure 1:**
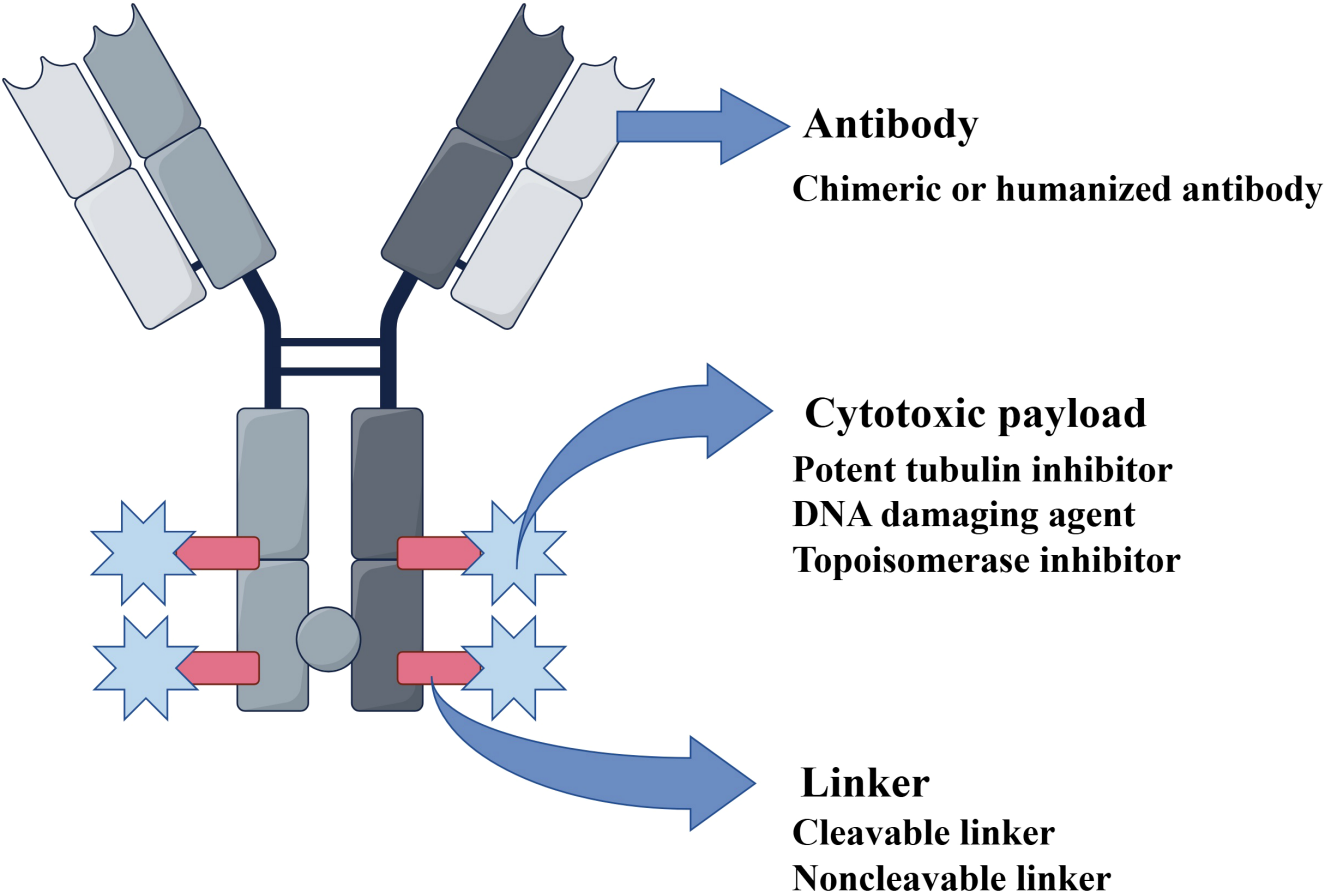
Structure of an antibody–drug conjugate (ADC). An ADC is structurally composed of three main components: 1. Antibody: A chimeric or humanized monoclonal antibody that specifically binds to a tumor-associated antigen. 2. Cytotoxic payload: A highly potent agent (e.g., tubulin inhibitor, DNA-damaging agent, or topoisomerase inhibitor). 3. Linker: A cleavable or non-cleavable chemical bridge that connects the antibody to the payload. Abbreviations: ADC, antibody–drug conjugate.

The advent of monoclonal antibodies (mAbs) has reformed cancer treatment by enabling precise targeting of tumor antigens (6). However, mAb monotherapy has often shown limited efficacy compared with conventional chemotherapy in some cases. Advances in biopharmaceutical technology have led to the rise of antibody–drug conjugates (ADCs), a new class of anticancer agents that combine the specificity of antibodies with the efficiency of cytotoxic drugs. This innovation has opened a new era in oncology, yielding distinct clinical achievements. An ADC consists of three key parts: an mAb, a cytotoxic payload, and a chemical linker (**Figure 1**). The mAb directs the ADC to specific antigens on target cells. This mechanism enables the precise delivery of the cytotoxic agent with minimal off-target effects on healthy tissues. The payload is a potent cytotoxic drug that kills the targeted cells. The linker serves as a stable connection between the antibody and the payload, ensuring that the cytotoxic agent remains attached during circulation. Without a well-designed linker, the payload may be prematurely released, leading to systemic toxicity and damage to normal tissues (7).

**Figure 2:**
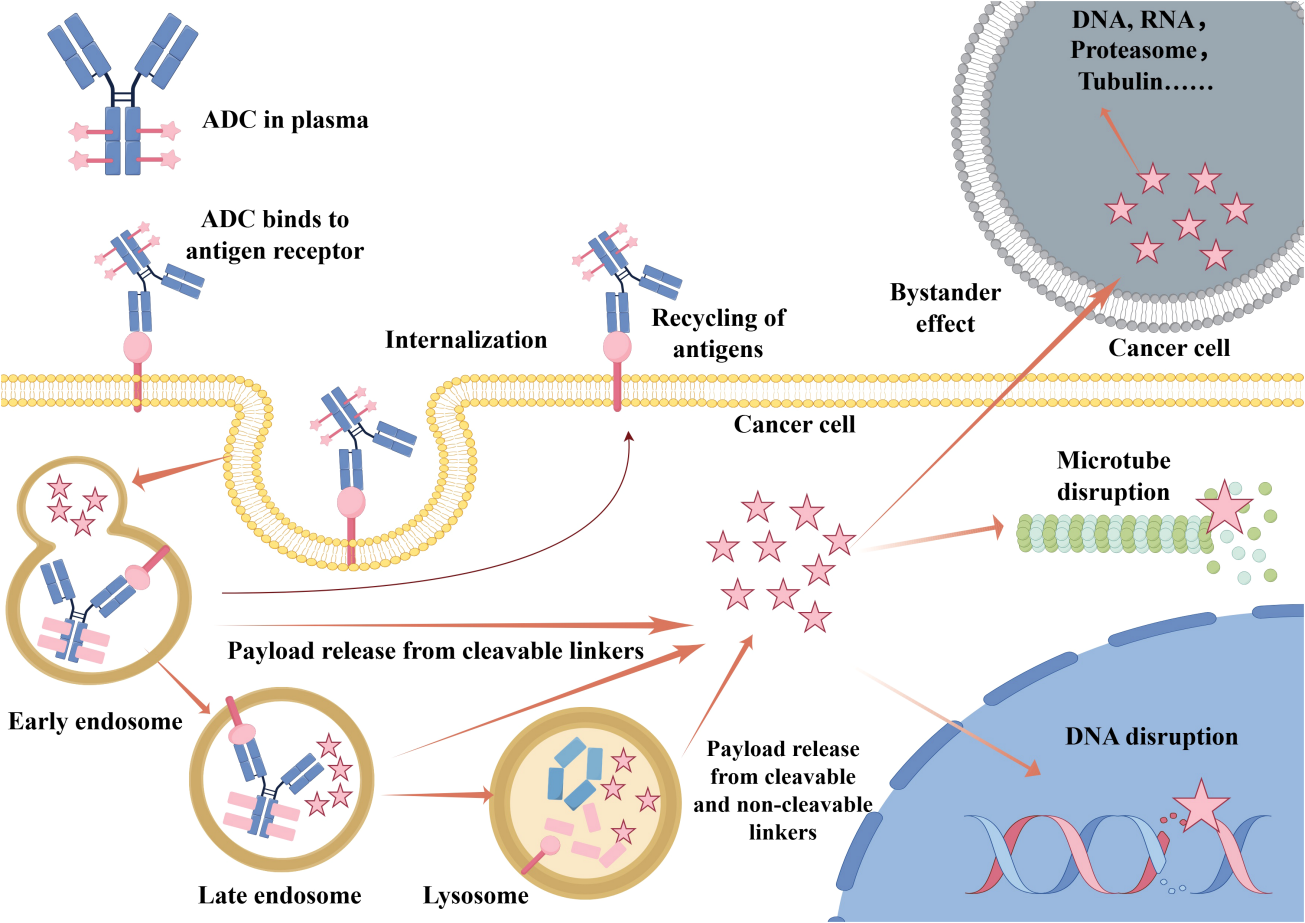
Mechanism of action of an antibody–drug conjugate (ADC). Upon administration, the ADC circulates in the plasma and specifically binds to target antigens on the tumor cell surface. The ADC–antigen complex is internalized via endocytosis and traffics through early endosomes, late endosomes, and finally lysosomes. The linker is cleaved and then the cytotoxic payload is released within the lysosome. The payload can disrupt critical cellular processes such as microtubule assembly or DNA integrity, leading to apoptosis. In some cases, the released payload may diffuse across the membrane to kill adjacent antigen-negative cells, which is known as the “bystander effect”. Some antigens may also undergo recycling back to the cell surface. Abbreviations: ADC, antibody–drug conjugate.

ADCs selectively kill cancer cells that express the target antigen. Upon binding to the antigen on the tumor cell surface, the ADC–antigen complex is internalized via endocytosis. It then traffics through endosomal compartments and ultimately fuses with lysosomes (8). Within the lysosome, enzymatic or chemical cleavage of the linker releases the cytotoxic payload (9, 10). The freed payload can then disrupt critical cellular processes—such as DNA replication or microtubule assembly—resulting in tumor cell death (**Figure 2**).

Furthermore, some released payloads are membrane-permeable and can diffuse into the tumor microenvironment. This ability to kill neighboring cancer cells that do not express the target antigen is referred to as the “bystander effect” (11–13). This phenomenon enhances ADC efficacy against heterogeneous tumors, which contain mixed populations of antigen-positive and antigen-negative cells. However, the bystander effect also raises the possibility of off-target toxicity. Achieving an optimal balance between therapeutic benefit and potential risk remains a crucial consideration in ADC development.

Since the U.S. Food and Drug Administration (FDA) approved Mylotarg^®^ (gemtuzumab ozogamicin) in 2000 for the treatment of acute myeloid leukemia (AML) (14), a total of 18 ADCs had received approval worldwide as of mid-2025 (**Table 1**). Furthermore, more than 100 ADC candidates are currently in various stages of clinical development, over 80% of which are being evaluated in solid tumors (15–17). Approved ADCs are already being used in the treatment of lung cancer, breast cancer, and other malignancies (18, 19). With expanding molecular targets and clinical indications, ADCs are ushering in a new era of targeted anticancer therapy. Notably, ADCs can remain effective even in cases where resistance to other targeted agents has developed, and they also provide opportunities to target novel antigens (20). For example, sacituzumab govitecan (Trodelvy^®^), which targets trophoblast cell-surface antigen 2 (TROP-2), has emerged as an important therapeutic option for advanced triple-negative breast cancer (21).

**Table 1:** Selected ADCs approved for marketing worldwide (as of mid-2025).

Brand name	Generic name	Company	Target	Payload/toxin	Linker type	Key indication(s)	First approved and status
Mylotarg	Gemtuzumab ozogamicin	Pfizer	CD33	Calicheamicin	Cleavable (hydrazone)	Acute myeloid leukemia (AML)	2000 accelerated approval 2010 voluntarily withdrawn due to safety concerns and lack of survival benefit in confirmatory trial. 2017 reapproved with new dosing schedule for a specific patient population.
Adcetris	Brentuximab vedotin	Seagen (now Pfizer)/Takeda	CD30	MMAE (Monomethyl auristatin E)	Protease-cleavable (valine-citrulline)	Hodgkin lymphoma, systemic anaplastic large cell lymphoma	2011 approved standard of care for several CD30-positive lymphomas
Kadcyla	Trastuzumab emtansine	Genentech/Roche	HER2	DM1 (maytansinoid derivative)	Non-cleavable (MCC)	HER2-positive breast cancer	2013 approved first ADC for solid tumors and first approved for adjuvant treatment
Besponsa	Inotuzumab ozogamicin	Pfizer	CD22	Calicheamicin	Cleavable (hydrazone)	Relapsed or refractory B-cell precursor acute lymphoblastic leukemia (ALL)	2017 approved
Lumoxiti	Moxetumomab pasudotox	AstraZeneca	CD22	PE38 (Pseudomonas exotoxin)	Non-cleavable (chemical bond)	Hairy cell leukemia (HCL)	2018 approved
Polivy	Polatuzumab vedotin	Genentech/Roche	CD79b	MMAE (monomethyl auristatin E)	Protease-cleavable (valine-citrulline)	Diffuse large B-cell lymphoma (DLBCL)	2019 approved
Padcev	Enfortumab vedotin	Astellas/Seagen	Nectin-4	MMAE (monomethyl auristatin E)	Protease-cleavable (valine-citrulline)	Locally advanced or metastatic urothelial carcinoma	2019 accelerated approval
Enhertu	Trastuzumab deruxtecan	Daiichi Sankyo/AstraZeneca	HER2	Deruxtecan (DXd, topoisomerase I inhibitor)	Protease-cleavable (tetrapeptide)	HER2-positive breast cancer, HER2-low breast cancer, gastric cancer, non-small cell lung cancer (NSCLC)	2019 approved known for its “bystander effect” and redefining HER2-targeted therapy
Trodelvy	Sacituzumab govitecan-hziy	Gilead	TROP-2	SN-38 (topoisomerase I inhibitor)	Cleavable (CL2A)	Triple-negative breast cancer (TNBC), urothelial carcinoma	2020 accelerated approval known for its “bystander effect”
Blenrep	Belantamab mafodotin-blmf	GSK	BCMA	MMAF (monomethyl auristatin F)	Non-cleavable (MC)	Relapsed or refractory multiple myeloma	2020 accelerated approval known for its “bystander effect” 2022 US approval withdrawn; available in other markets
Zynlonta	Loncastuximab tesirine-lpyl	ADC Therapeutics	CD19	PBD dimer (pyrrolobenzodiazepine dimer)	Cleavable (valine-alanine)	Large B-cell lymphoma	2021 approved
Tivdak	Tisotumab vedotin-tftv	Genmab/Seagen	Tissue factor (TF)	MMAE (monomethyl auristatin E)	Protease-cleavable (valine-citrulline)	Recurrent or metastatic cervical cancer	2021 accelerated approval
Elahere	Mirvetuximab soravtansine-gynx	ImmunoGen (now GSK)	FRα (foliate receptor alpha)	DM4 (maytansinoid derivative)	Cleavable (SPDB)	Platinum-resistant ovarian cancer	2022 accelerated approval
Akalux	Cetuximab sarotalocan sodium	Rakuten Medical	EGFR	IR700 (photosensitizer)	Non-cleavable (chemical bond)	Head and neck cancer	2020 approved in Japan A photodynamic therapy (PDT) conjugate.
Aidixi	Disitamab vedotin	RemeGen	HER2	MMAE (monomethyl auristatin E)	Protease-cleavable	Gastric cancer, breast cancer, urothelial carcinoma	2021 approved in China
Tuo Da Wei	Sacituzumab tirumotecan	Kelun-Biotech/Merck & Co. (MSD)	TROP-2	Topoisomerase I inhibitor	Cleavable (proprietary)	Triple-negative breast cancer (TNBC)	2024 approved in China
Datroway	Datopotamab deruxtecan	AstraZeneca/Daiichi Sankyo	TROP-2	Dxd (topoisomerase I inhibitor)	Cleavable (tetrapeptide)	HR+/HER2-breast cancer, NSCLC	Jan 2025 approved Jun 2025 accelerated approval for NSCLC
Emrelis	Telisotuzumab vedotin	AbbVie	c-Met	MMAE(Monomethyl auristatin E)	Protease-cleavable	c-Met high NSCLC	May 2025 (accelerated)

ADCs have begun to transform cancer treatment in recent years. However, their clinical translation requires addressing multifaceted challenges, including pharmacokinetic complexity, target antigen heterogeneity, controlled payload release, linker stability, toxicity, structural optimization, and resistance mechanisms (22, 23). Numerous studies are underway to tackle these issues. ADCs are expected to become a viable alternative to conventional chemotherapy in the future. In this review, we review the structure of ADCs and their molecular mechanisms of action, briefly summarize the advantages and limitations of approved ADC therapeutics, discuss existing challenges and strategies for achieving optimal efficacy, outline the prospects of novel ADCs under development, and examine the current landscape of combination therapies between ADCs and immune checkpoint inhibitors (ICIs).

## Components and mechanisms of ADCs

### Antibodies in ADCs

The ideal antibody should exhibit a high affinity for the target antigen, facilitate efficient internalization, demonstrate low immunogenicity, and have a long plasma half-life. High immunogenicity can cause the antibody to be recognized and destroyed by the body’s immune system before it can reach the target, reducing its efficacy (24). In the early days of antibody therapy, murine antibodies were commonly used. However, they were cleared rapidly from the bloodstream due to their high immunogenicity in humans, which led to serious side effects (25). With the emergence of recombinant DNA technology, murine antibodies have largely been replaced by less immunogenic chimeric and humanized antibodies (26).

For antibodies targeting tumor cells, efficacy largely depends on their binding affinity for the surface antigen. Generally, a higher affinity promotes more rapid internalization (27). However, excessively high affinity can hinder penetration into solid tumors. Treating solid tumors is more complex than treating hematological malignancies due to the “binding site barrier (BSB)” effect (26). Therefore, an optimal level of affinity must be determined to balance efficient tumor cell internalization with adequate tissue penetration and anticancer potency.

Antibody size is another critical factor influencing tumor penetration. The large molecular weight of immunoglobulin G (IgG) antibodies (approximately 150 kDa) often impedes their diffusion through capillary walls and the dense extracellular matrix of tumor tissues (28). In contrast, smaller antibody formats (e.g., scFv, Fab) not only retain high affinity and specificity but also penetrate tumors more effectively, thereby significantly enhancing antitumor efficacy (29). A trade-off of this size reduction, however, is a shorter *in vivo* half-life. Consequently, multiple factors must be carefully balanced when designing ADCs incorporating these miniaturized antibodies.

**Figure 3:**
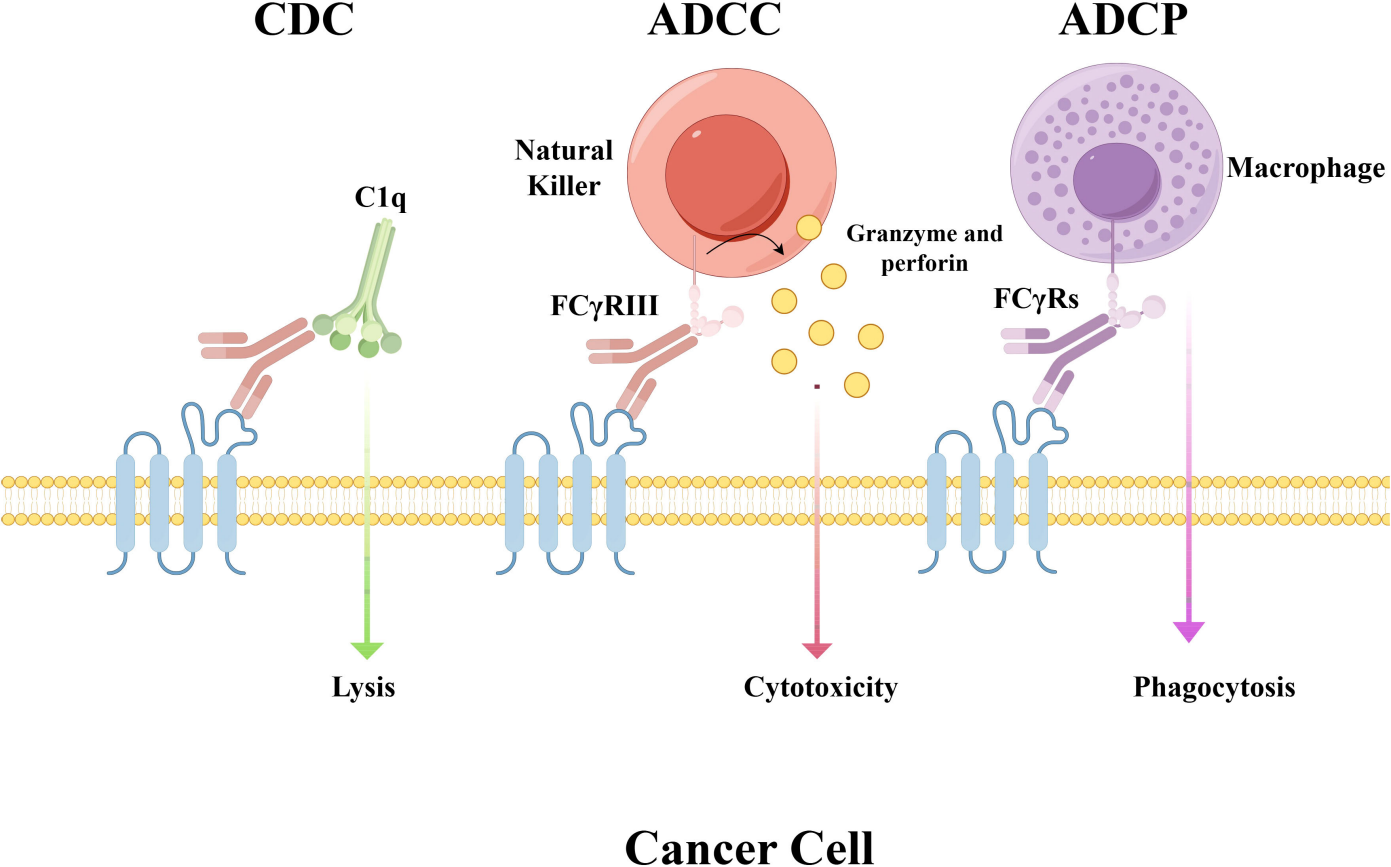
Mechanisms of Fc-mediated effector functions. This schematic illustrates antibody-dependent immune responses mediated through Fc gamma receptors (FcγRs). CDC: complement cascade activation and consequent cell lysis initiated by antibody-antigen binding ADCC: engagement of FcγRs (e.g., FcγRIII) on natural killer (NK) cells induces the release of cytolytic molecules such as granzyme and perforin, resulting in target cell death. ADCP: FcγRs on macrophages mediate this process, which enables the engulfment and damage of target cells. Abbreviations: FcγRs, Fc gamma receptors; CDC, complement-dependent cytotoxicity; ADCC, antibody-dependent cellular cytotoxicity; ADCP, antibody-dependent cellular phagocytosis.

The antibodies currently used in ADCs are mostly IgG, which includes four subtypes: IgG1, IgG2, IgG3, and IgG4. The choice of IgG subclass primarily determines the effector functions and immunogenicity risk of the antibody component (30). Among the IgG subtypes, IgG1 is the dominant choice (found in 85% of clinical-stage ADCs) due to its superior Fcγ receptor (FcγR) binding capacity and extended serum half-life (14–21 days) (31, 32). IgG1 possesses strong effector functions, mediated through antibody-dependent cell-mediated cytotoxicity (ADCC), antibody-dependent cellular phagocytosis (ADCP), and complement-dependent cytotoxicity (CDC), due to its high binding affinity for FcγRs (**Figure 3**) (33). When a target is highly and specifically overexpressed on tumor cells with minimal expression in normal tissues (e.g., HER2), selecting the IgG1 isotype leverages its potent effector functions (34). This activates the immune system to attack the tumor, complementing the payload’s cytotoxic effect and resulting in a multi-mechanistic killing strategy. However, IgG1 may also induce ADCC/ADCP effects against normal cells with low target expression, leading to on-target, off-tumor toxicity (35). Furthermore, by binding to FcγRs on immune cells, IgG1 may be internalized, causing unintended payload release and resulting in off-target toxicities such as myelosuppression (e.g., neutropenia) (36).

IgG2 has minimal effector function due to its low affinity for most FcγRs and is a poor inducer of ADCC, ADCP, and CDC (37). However, a disadvantage of IgG2 is its structural complexity. The hinge region forms a unique, rigid structure stabilized by multiple disulfide bonds, a feature that can lead to product heterogeneity through the formation of multiple isoforms (38). IgG2 is selected for ADCs when the desired activity must rely entirely on the payload’s cytotoxicity to avoid risks associated with Fc effector functions. This is particularly relevant for targets that have essential physiological functions or are expressed at low levels in normal tissues, as this choice minimizes the risk of “on-target, off-tumor” toxicity mediated by effector functions (39, 40).

Among all human IgG subclasses, IgG3 has the most potent effector functions. It exhibits the highest affinity for FcγRIIIa and the greatest capacity to activate CDC (30). However, its core drawback is an extremely short half-life (approximately 7 days) resulting from its low affinity for FcRn. The key amino acid sequence in IgG3’s FcRn binding interface (e.g., H435) differs from that of other subtypes, resulting in significantly weaker binding at acidic pH. Consequently, IgG3 is more efficiently sorted to lysosomes for degradation rather than being recycled, leading to its rapid clearance. A key structural feature of IgG3 is its exceptionally long hinge region (approximately 62 amino acids), which is rich in cysteine and proline residues that form a complex structure (41). While this unique architecture confers exceptional flexibility and potent effector functions, it also renders the antibody highly susceptible to proteolytic cleavage by enzymes such as neutrophil elastase and matrix metalloproteinases (MMPs). This cleavage results in fragmentation into Fab and Fc segments in the bloodstream, further accelerating its clearance (37). This short half-life is a critical weakness for ADCs, which require prolonged circulation for optimal tumor distribution. Coupled with its inherent instability, these pharmacokinetic and physicochemical defects explain why IgG3 is rarely used in ADC development.

IgG4 has low affinity for FcγRs, resulting in negligible effector functions and an inability to activate CDC. Native IgG4 undergoes “Fab-arm exchange” *in vivo*. In this process, a half-molecule (one heavy chain and one light chain) from one IgG4 exchanges with a half-molecule from another IgG4 that has a different specificity, resulting in a bispecific antibody (42). This dynamic exchange is particularly problematic for ADCs because it results in a loss of targeting specificity—producing ineffective antibodies that fail to bind the target—or, worse, generates bispecific antibodies (BsAbs) that engage both tumor and healthy tissues, potentially causing severe off-target toxicity (43). The introduction of a point mutation (S228P; serine to proline) in the hinge region significantly stabilizes the IgG4 structure and prevents Fab-arm exchange (44). This stabilization is critical, and as such, all therapeutic antibodies based on the IgG4 isotype must incorporate this modification. When minimal effector function is desired, the engineered, stabilized IgG4 (IgG4-S228P) serves as a commonly used inert scaffold (45).

Modern ADC design is no longer limited to natural subclasses but employs genetic engineering techniques to modify the Fc region for precise control of effector functions, offering greater flexibility in IgG subclass selection (46). Fc silencing is the most common strategy, accomplished by introducing point mutations into an IgG1 backbone—chosen for its superior stability and long half-life (47, 48). Mutations such as L234A/L235A and P329G (termed the “LALA-PG” mutation) effectively abrogate binding to FcγRs and C1q (49). This approach retains the favorable stability, long half-life, and low immunogenicity risk of IgG1 while eliminating effector functions. It has become the platform of choice for many new-generation ADCs (e.g., T-DXd) (50), ensuring that efficacy stems solely from targeted payload delivery, thereby preventing Fc-mediated, off-target toxicity. Conversely, Fc enhancement involves introducing mutations (e.g., S239D/I332E) to increase affinity for FcγRIIIa, potently boosting ADCC (51). This strategy is employed when potent immune activation is desired to complement the ADC’s payload-mediated killing. However, it requires careful evaluation of on-target, off-tumor toxicity risks.

In summary, engineered Fc-silenced IgG1 is becoming the platform of choice in ADC development, as it merges the favorable pharmacokinetics (PK) of IgG1 with a superior safety profile devoid of effector function. This contrasts with the selection of native IgG1 or IgG4, which necessitates a careful, case-specific trade-off based on the target biology and mechanism of action.

### Payloads in ADCs

#### First-generation payloads: conventional chemotherapy agents

The early development of ADC technology took place from the 1990s to the early 2000s. First-generation payloads were composed of conventional chemotherapeutic agents like methotrexate, doxorubicin, and mitomycin (52). While their mechanisms of action (e.g., inhibiting DNA synthesis) were well-established, these drugs exhibited relatively low potency, with cytotoxicity typically in the micromolar (μM) IC50 range (53). Their high hydrophilicity hindered cell membrane penetration, preventing a bystander killing effect on adjacent antigen-negative cells and significantly limiting efficacy against heterogeneous tumors (54). Furthermore, as substrates for efflux pumps like P-glycoprotein (P-gp), they were prone to multidrug resistance (MDR) (55). Due to insufficient potency, poor stability, and a narrow therapeutic window, most first-generation ADCs failed in clinical trials. Nonetheless, these pioneering efforts validated the core ADC concept and underscored the urgent need for more potent warheads.

#### Second-generation payloads: highly potent tubulin inhibitors

The maturation and regulatory approval of ADC technology spanned from the 2000s to the mid-2010s. This era was characterized by two potent payload classes: auristatins (e.g., MMAE, MMAF) and maytansinoids (e.g., DM1, DM4) (56, 57). These agents were 100 to 1,000 times more potent than their first-generation predecessors, with activity in the nM to pM range, ensuring tumor cell killing even with low ADC internalization (58). Their development was enabled by advanced linker technologies. For instance, MMAE-based ADCs used protease-cleavable linkers (Val-Cit) for stable circulation and targeted payload release in the lysosome. The released MMAE, due to its membrane permeability, mediates a potent bystander effect by diffusing into and killing adjacent antigen-negative cells—a key advantage for solid tumors (59). Conversely, MMAF is charged and hydrophilic, trapping it inside the original cell and eliminating this effect (60, 61). Second-generation ADCs yielded breakthrough drugs like Adcetris^®^ and Kadcyla^®^ (which features the maytansinoid DM1). A key limitation of these tubulin-inhibiting payloads was their primary efficacy against dividing cells, often sparing quiescent populations. Some also remained susceptible to efflux-mediated MDR (**Table 2**).

**Table 2:** Comparison of ADC payloads.

Feature dimension	First-generation payloads	Second-generation payloads	Third-generation and emerging payloads
Representative type	Conventional chemotherapy drugs	Microtubule/tubulin inhibitors	DNA—damaging agents, topoisomerase inhibitors, immunostimulators, RNA-targeting agents
Primary mechanism	Interference with DNA synthesis or function	Disruption of microtubule dynamics, inhibition of mitosis	Induction of DNA double-strand breaks, topoisomerase inhibition, immune activation, RNA interference
Key characteristics	Broad mechanism; limited potency; no bystander effect	High potency (nanomolar level); exhibits bystander effect (e.g., MMAE)	Ultra-high potency (picomolar level); potent bystander effect; effective in both dividing and non-dividing cells
Limitations	Low therapeutic index; off-target toxicity; susceptibility to resistance mechanisms	Cytotoxicity limited to dividing cells; potential for on-target peripheral toxicity	Complex synthesis; narrow therapeutic window; potential for novel resistance mechanisms
Examples	Methotrexate, doxorubicin	DM1, DM4, MMAE, MMAF	DXd, calicheamicin, PBD dimers, duocarmycins, TLR agonists, STING agonists

#### Third-generation payloads: DNA-damaging agents and emerging mechanisms

The breakthrough and innovation phase of ADC technology began in the late 2010s and continues to the present. Third-generation payloads include DNA topoisomerase I inhibitors, such as DXd (deruxtecan) and SN-38, and pyrrolobenzodiazepine (PBD) dimers like talirine. DXd is a revolutionary representative, serving as the payload for the third-generation ADC drug Enhertu^®^ (DS-8201) (62, 63). PBD dimers exhibit extremely potent DNA cross-linking capabilities. Although calicheamicin was discovered earlier (used in Mylotarg^®^), its powerful DNA-cleaving mechanism aligns closely with the principles of third-generation payloads (64). These payloads demonstrate even higher potency than second-generation agents, particularly DNA-damaging agents, with IC50 values reaching the picomolar (pM) level. Their mechanism of action shifted from targeting microtubules to directly damaging DNA, enabling them to kill both actively dividing and quiescent cells, thereby broadening their therapeutic scope (65). Their strong membrane permeability contributes to a pronounced bystander effect, which is critical for overcoming tumor heterogeneity and acquired resistance. Many DNA-damaging agents are not substrates of P-gp efflux pumps, allowing them to remain effective against multidrug-resistant tumor cells (66). The third-generation concept also extends beyond cytotoxic payloads to include immune-stimulating agents (e.g., TLR agonists), designed to activate local immune responses rather than directly kill cells (67).

### Characteristics of an ideal payload

#### High potency

The payload must possess extremely high cytotoxicity, typically in the pM to nM range. The antibody’s payload capacity is limited, typically with a drug-to-antibody ratio (DAR) of 2–4. Furthermore, only a small fraction of the administered ADC molecules are ultimately internalized into tumor cells (68). Consequently, each internalized payload molecule must be potent enough to kill a tumor cell, or even multiple cells via the bystander effect. The IC_50_ values of auristatins (e.g., MMAE) and exatecan derivatives (e.g., DXd) are in the pM–nM range, far exceeding the potency (by orders of magnitude) of traditional chemotherapy drugs, which are typically in the micromolar (μM) range (62).

#### Well-defined mechanism of action

The payload must have a well-defined and highly efficient mechanism of action capable of rapidly inducing cell death. A clear mechanism of action helps predict its efficacy, potential drug resistance, and toxic side effects. Inhibitors of tubulin polymerization (e.g., MMAE, DM1) prevent mitosis, leading to cell-cycle arrest and apoptosis (63). DNA-damaging agents (e.g., DXd, PBDs, and calicheamicin) cause DNA double-strand breaks—the most lethal type of DNA damage—which are effective against both dividing and quiescent cells (64). The payload DXd in DS-8201 (Enhertu) is a topoisomerase I inhibitor that exerts cytotoxicity by preventing DNA relegation (63).

#### Optimal hydrophilic–lipophilic balance

The payload must exhibit sufficient hydrophilicity to prevent excessive hydrophobicity. Excessive hydrophobicity (a high Log P value) promotes ADC aggregation *in vivo*, rapid clearance by the immune system, increased hepatic toxicity, and unfavorable PK (69). Conversely, for the treatment of solid tumors, the payload requires a degree of hydrophobicity to enable cell membrane penetration and to mediate the bystander effect, thereby killing adjacent tumor cells with low or no target expression (70). Therefore, a critical balance must be struck between hydrophilicity (which ensures ADC solubility and stability) and moderate hydrophobicity (which ensures membrane permeability and bystander killing). The comparison between auristatins MMAE and MMAF exemplifies this trade-off: MMAF, which features a terminal phenylalanine carboxylate group, is more hydrophilic than MMAE but consequently exhibits a minimal bystander effect due to its impaired membrane permeability (71).

#### Stability in circulation and efficient release in target cells

The payload must remain highly stable in the bloodstream after conjugation but be efficiently released upon internalization into target cells. Premature dissociation of the payload from the antibody in circulation can lead to severe off-target toxicity in healthy tissues (e.g., bone marrow, gastrointestinal tract) and is a primary driver of dose-limiting toxicities for ADCs (72). The payload is only active upon its specific release inside tumor cells. This efficacy is critically dependent on the linker design. Cleavable linkers (e.g., peptide linkers) are engineered to remain inert in plasma but undergo hydrolysis in the acidic environment of lysosomes or via enzymatic cleavage by specific proteases (e.g., cathepsin B, β-glucuronidase) (73). A prominent example is the tetrapeptide-based linker (GGFG) used in trastuzumab deruxtecan (T-DXd, Enhertu). This ADC demonstrates high stability in plasma due to its robust linker, minimizing off-target release. The active payload (DXd) is liberated specifically within tumor cells following cleavage by lysosomal proteases, ensuring potent and targeted cytotoxicity while sparing healthy tissues (23).

#### Low susceptibility to MDR

An ideal payload should avoid being a substrate for efflux pump proteins such as P-gp. Tumor cells often overexpress these pumps, which mediate the efflux of traditional chemotherapeutic agents (e.g., paclitaxel) from the cell, conferring drug resistance (74). If the ADC payload is also a substrate, it can be effluxed, leading to reduced intracellular concentration and treatment failure. Many tubulin inhibitors (e.g., MMAE and DM1) are vulnerable to MDR mechanisms. In contrast, certain DNA-damaging agents such PBD dimers are poor substrates for these pumps and can effectively circumvent this form of resistance (75). Therefore, developing payloads that evade efflux pumps is a critical design goal. For example, Mabwell Bioscience’s novel ADC, 7MW4911, utilizes a proprietary topoisomerase I inhibitor payload (MF-6). In preclinical studies, 7MW4911 demonstrated superior antitumor activity compared with MMAE- or DXd-based ADCs in models with ABC transporter-mediated MDR, highlighting its potential to overcome this major clinical challenge (76).

### Linkers in ADCs

Linkers can be categorized into two main classes: cleavable (degradable) and non-cleavable (stable). Cleavable linkers take advantage of the environmental differences between the systemic circulation and tumor cells to accurately release the free cytotoxic drug. Cleavable linkers can be further categorized into chemically cleavable linkers, such as hydrazone bonds and disulfide bonds, and enzymatically cleavable linkers, such as glucuronide bonds and peptide bonds (14).

Hydrazone is a typical acid-sensitive linker. Hydrazone-linked ADCs are usually stable in the bloodstream but are hydrolyzed to release the cytotoxic payload in lysosomes and endosomes after internalization into target tumor cells (77). However, hydrolysis of the hydrazone bond is not completely confined to lysosomes, and occasional hydrolysis can also occur in the plasma, leading to reduced targeting efficiency and off-target effects (78). So far, ADCs containing hydrazone linkers have been used primarily in hematologic malignancies. For example, both gemtuzumab ozogamicin and inotuzumab ozogamicin use a hydrazone linker to conjugate calicheamicin to antibodies for the treatment of AML and acute lymphoblastic leukemia (ALL), respectively. The disulfide bond linker is another type of chemically cleavable linker, sensitive to reductive glutathione (GSH) (79). The concentration of GSH in the blood is considerably lower than the intracellular concentration in tumor cells (80). Therefore, disulfide bond linkers remain stable in the circulatory system while selectively releasing active payloads in tumor cells with elevated GSH levels.

As far as enzyme-sensitive linkers are concerned, peptide linkers are sensitive to lysosomal proteases and have been employed in many ADCs (81). Lysosomal proteases, such as cathepsin B, are usually overexpressed in tumor cells, enabling accurate drug release in the vicinity of the tumor. However, due to the presence of protease inhibitors in the blood, these linkers are generally stable in the bloodstream and help reduce the risk of side effects (82).

Among the approved ADC drugs, most utilize peptide linkers. For example, brentuximab vedotin uses a valine–citrulline linker. Beta-glucuronide linkers represent another type of enzyme-sensitive linker commonly used in ADCs. They can be cleaved by beta-glucuronidase in tumor cells to release the payload (83). Non-cleavable linkers are inert to common chemical and enzymatic conditions *in vivo*, such as thioether or maleimidocaproyl groups. These linkers exhibit high stability under various physiological conditions, including the tumor microenvironment’s pH, enzymatic activity, or reducing agents (84). After an ADC with a non-cleavable linker enters the tumor cell via target-mediated endocytosis, it is internalized into an endosome, which later fuses with a lysosome. Within the lysosome, the antibody backbone is extensively degraded by abundant proteases (e.g., cathepsins) and peptidases into small amino acids or short peptides. At this stage, the payload—which remains covalently attached to an amino acid residue (typically lysine or cysteine) via the non-cleavable linker—is released as a consequence of antibody degradation (85). However, the released entity is a payload derivative still connected to the linker and an amino acid remnant. The payload is considered “released” only after the hydrolysis of the specific antibody amino acid residue to which it is bound.

In the development of ADCs, the selection of an appropriate linker remains a major challenge. The ideal linker must be stable in the bloodstream while enabling rapid and effective release of the payload within tumor cells. As ADC technology continues to advance, the design and optimization of linkers will play a central role in driving the development of novel ADC therapeutics, ultimately improving treatment efficacy and reducing adverse effects.

### Conjugation methods

In addition to the selection of the antibody, linker, and payload, the method used to conjugate the small-molecule moiety (i.e., the linker–payload complex) to the antibody is also critical for the successful construction of ADCs. Random conjugation remains a technology employed in some approved drugs. In lysine conjugation, the ϵ-amino groups of the abundant lysine residues on the antibody surface (typically 80–100 lysines, with 10–20 being reactive) react with activated esters (e.g., NHS esters) on the linker–payload (86). Because the conjugation occurs randomly, this process generates a heterogeneous mixture of ADC molecules with a wide range of DARs, such as DAR 0, 2, 4, 6, 8, or even higher (87). Although the resulting amide bonds are chemically stable, conjugation at sites within hydrophobic regions can promote ADC aggregation, whereas modification in the antigen-binding domain may impair antigen binding (88).

In cysteine conjugation, the four interchain disulfide bonds in the antibody hinge region are partially reduced to generate eight free cysteine thiol groups. These thiols then undergo alkylation by electrophilic moieties (e.g., maleimide) on the linker–payload (89). A major limitation of this approach is the instability of the maleimide–thioether bond. In plasma, this bond is prone to thiol exchange reactions with sulfhydryl-bearing molecules such as albumin, leading to premature payload release and potential off-target toxicity (90). The inherent limitations of random conjugation have driven the advancement of ADC technologies toward site-specific conjugation strategies (**Table 3**).

**Table 3:** Comparison of ADC conjugation technologies.

Conjugation technology	Mechanism/site	DAR homogeneity	Stability and pharmacokinetics	Key advantages	Key disadvantages
Lysine conjugation	Non-specific conjugation to ϵ-amines of surface lysine residues.	Poor. Heterogeneous mixture with broad DAR distribution (0–8).	Low plasma stability; rapid clearance; variable exposure.	Simple process; no antibody engineering required.	Low batch consistency; narrow therapeutic window; potential activity loss.
Cysteine conjugation	Conjugation to thiols from reduced interchain disulfides.	Moderate. DAR 0/2/4/6/8 mix; may be purified to defined species.	Moderate. Risk of thiol-maleimide exchange; linker instability in plasma.	Higher homogeneity than lysine; industry-known process.	Potential aggregation; *in vivo* destabilization via retro-Michael reaction.
THIOMAB/engineered cysteine	Site-specific introduction of cysteines via mutation (e.g., Ser→Cys).	High. Homogeneous DAR2 or DAR4 possible.	High stability; PK comparable with native mAb; low clearance.	Optimal homogeneity; minimal aggregation; improved safety.	Requires protein engineering and new cell line development.
Transglutaminase	Enzymatic conjugation to glutamine via microbial transglutaminase (mTG).	High. Homogeneous DAR2 or DAR4 with peptide tag.	High; stable amide bond; favorable PK; low clearance.	Site-specific; no reducing agent needed; native bond.	May require tag insertion; enzyme activity variable.
Sortase	Enzymatic cleavage of C-terminal LPXTG motif followed by glycyl coupling.	High. Site-specific, typically DAR2.	High; peptide bond highly stable; PK profile favorable.	Genetic encoding possible; C-terminal specificity.	Low reaction efficiency; reversibility; not easily scalable.
Glycoengineering/EndoS2	Fc glycan trimming (to GlcNAc) followed by chemoenzymatic conjugation.	High. Homogeneous DAR2 via native glycosylation site.	High; PK similar to native antibody; low immunogenicity.	Uses natural site; no sequence modification.	Only for IgG1; DAR limited to 2; requires glycosylation.
Aldehyde tag/oxime ligation	Formylglycine (fGly) formation by FGE; conjugation via oxime bond.	High. Homogeneous DAR2 or DAR4.	High; oxime bond stable *in vivo*; excellent PK.	Genetically encoded; strong bioorthogonality.	Co-expression of FGE required; may affect folding.
Unnatural amino acid (uAA)	Incorporation of azide-/alkyne-bearing uAA via genetic code expansion.	Very high. Perfectly homogeneous DAR2 or DAR4.	Extremely high; triazole bond very stable; optimal PK.	Absolute specificity; bioorthogonal; no natural residue interference.	Low yield; high cost; complex cell line and process development.
Photo-induced conjugation	UV activation of benzophenone probes for C–H bond insertion.	Low to moderate. Limited control over site selectivity.	Moderate; C–C bond stable, but UV may cause damage.	No pre-engineered antibody; broad applicability.	UV may denature Ab; challenging control and scalability.
Metal complex-mediated	Chelator conjugated to lysines; metal ion (e.g., Cu²^+^) bridges payload.	Poor. Heterogeneous in both chelator and metal loading.	Poor; metal transchelation *in vivo* causes premature release.	Modular; suitable for imaging probes.	Unsuitable for therapeutics; instability and toxicity risks.
Affinity peptide-mediated	Non-covalent high-affinity peptide binding followed by covalent trapping.	High. Can achieve DAR1 or DAR2.	Dependent on peptide–linker stability.	Modular; “plug-and-play” payload system.	Risk of immunogenicity from exogenous peptide motifs.

The engineered cysteine technology utilizes genetic engineering to replace specific amino acid residues (typically serine) at defined sites on the antibody (such as the native cysteine pairs on the heavy chain) with cysteine, thereby introducing new reactive thiol groups (–SH). These thiol groups can specifically react with linker–payload constructs containing functional moieties such as maleimide (91). Since the number of introduced thiol groups is precisely controlled (usually 2 or 4), all ADC molecules carry the same drug load, resulting in a highly homogeneous DAR (87). Furthermore, because the conjugation sites are engineered to avoid labile regions (e.g., the hinge region) and hydrophobic patches, the conjugate exhibits improved stability (92). The highly uniform DAR and enhanced stability significantly reduce clearance in systemic circulation, prolong the half-life, and ultimately contribute to prolonged efficacy and reduced systemic toxicity.

The microbial transglutaminase (mTG) technology utilizes mGT to specifically recognize the acyl donor glutamine (Q295) on the Fc region of antibodies and catalyze a cross-linking reaction between its amide group and a linker–payload construct containing a primary amine group (e.g., derived from a lysine side chain) (93). Alternatively, a “glutamine tag” (Q-tag) can be genetically introduced into specific antibody sequences to provide an enzymatic recognition site. The high specificity of the enzyme ensures that conjugation occurs exclusively at predetermined sites, resulting in nearly all ADC molecules carrying two payloads (due to the two symmetric Fc regions in IgG1) and achieving a highly uniform DAR 2 (94). This technology forms a stable amide bond that is resistant to chemical and enzymatic degradation in plasma, thereby minimizing the risk of cleavage and reducing the propensity for aggregation. The homogeneous molecular population and stable linkage promote consistent *in vivo* behavior, leading to more predictable PK and reduced interindividual variability (95).

The non-canonical amino acid (nnAA) technology involves the site-specific introduction of a nnnAA (such as *para*-acetylphenylalanine, pAcF) into the antibody sequence. This nnAA contains unique reactive functional groups (e.g., a ketone moiety) absent in natural amino acids, enabling highly efficient and specific conjugation to the linker–payload via bioorthogonal reactions such as oxime ligation (96). By controlling the number of incorporated nnAA residues, DAR can be precisely adjusted, allowing for the generation of ADCs with defined DAR values (e.g., DAR 2, 4, 6, etc.) (87). The stability of the resulting conjugate bond depends on the specific conjugation chemistry employed. For instance, linkages formed via oxime chemistry (analogous to those used in aldehyde tag approaches) may display reduced stability under acidic conditions relative to amide bonds (97).

The glycan remodeling/EndoS2 technology leverages the fact that antibodies are glycosylated proteins bearing a conserved N-glycan on their Fc region. The process begins by treating the antibody with an endoglycosidase (such as EndoS2) to remove the native glycan structures, leaving only a core N-acetylglucosamine (GlcNAc) residue on the Fc segment (98). An engineered glycosyltransferase (e.g., GalT/Y289L) then specifically attaches a sugar derivative (such as a UDP-sugar analog) carrying a linker–payload to this GlcNAc residue. Since each antibody Fc region contains a single conserved N-glycan site, the resulting ADC primarily exhibits a DAR of 2 (99). The newly formed glycosidic bond is natural in origin and demonstrates high stability in plasma, with strong resistance to hydrolysis. Moreover, because glycosylation is native to antibodies and the conjugation occurs within the Fc region, the antigen-binding function remains unaltered, enabling the ADC to retain a long circulation half-life comparable with that of unconjugated antibodies.

The aldehyde tag/oxime chemistry approach involves the genetic introduction of a short peptide sequence (“aldehyde tag”, LCXPXR) at the terminus of the antibody (e.g., the C-terminus of the heavy chain). This sequence is recognized by the formylglycine-generating enzyme (FGE), which catalytically oxidizes the cysteine residue within the tag to generate a unique formylglycine (fGly) residue. fGly contains a reactive aldehyde group (–CHO), which subsequently undergoes specific oxime ligation with a linker–payload construct containing an aminooxy group (–ONH_2_) (100, 101). Each introduced tag yields one aldehyde group, enabling precise conjugation with a DAR of 2. However, while the oxime bond is relatively stable under acidic conditions, it may undergo reversible reactions at neutral to alkaline pH. *In vivo*, it can also be susceptible to chemical and enzymatic degradation, posing a potential risk of cleavage in plasma and premature payload release (102). This relative instability may adversely affect PK, leading to accelerated clearance. Nevertheless, the highly uniform DAR achieved using this strategy remains superior to that obtained with random conjugation methods.

In summary, the conjugation technology field is rapidly moving from random conjugation toward site-specific strategies to produce homogeneous ADCs with excellent stability and PK. Future efforts are exploring ultra-precise techniques like unnatural amino acid incorporation and modular platforms, although each emerging approach still faces many challenges.

## The ADCs approved for the market

ADCs have achieved remarkable clinical and commercial success, as demonstrated by the recent approval and strong market performance of agents such as trastuzumab deruxtecan (Enhertu^®^). These advancements have significantly influenced the pharmaceutical industry. According to industry reports, a total of 18 ADC drugs had received regulatory approval worldwide between 2000 and mid-2025. From the perspective of drug composition and technological features, the evolution of ADCs is commonly divided into three distinct generations (**Table 4**).

**Table 4:** Comparison of three generations of ADC technologies.

Feature	First-generation ADCs	Second-generation ADCs	Third-generation ADCs
Antibody	Murine or chimeric	Humanized	Fully human or engineered human
Linker	Acid-labile (e.g., hydrazone)	Protease-cleavable (e.g., Val-Cit, Val-Ala)	Enzyme-cleavable, sulfonate-based, self-immolative
Payload	Cytotoxic (e.g., calicheamicin)	Highly potent (e.g., MMAE, DM1)	Ultra-potent (e.g., Dxd, PBD)
Conjugation technology	Random (heterogeneous DAR)	Random (heterogeneous DAR)	Site-specific (homogeneous DAR)
Representative drugs	Mylotarg^®^ (gemtuzumab ozogamicin)	Adcetris^®^ (brentuximab vedotin), Kadcyla^®^ (ado-trastuzumab emtansine)	Enhertu^®^ (fam-trastuzumab deruxtecan), Trodelvy^®^ (sacituzumab govitecan)
Advantages	Proof of concept	Improved efficacy and safety over 1st gen	Precise therapy, wider therapeutic window, homogeneous DAR
Limitations	Immunogenicity, high toxicity, low efficacy	Off-target toxicity, limited bystander effect, heterogeneous DAR	Complex manufacturing, novel/unpredictable toxicities

### The first-generation ADCs

The first-generation ADCs, such as BR96-doxorubicin, gemtuzumab ozogamicin, and inotuzumab ozogamicin, employed non-cleavable linkers, traditional chemical cytotoxic payloads, and murine-derived antibodies (IgG4). Gemtuzumab ozogamicin (Mylotarg^®^) was the first ADC therapeutic approved for clinical use worldwide. It consists of a humanized monoclonal IgG4 antibody targeting CD33 and the cytotoxic agent N-acetyl-γ-calicheamicin, connected via a cleavable hydrazone linker (31). Mylotarg received initial approval in 2000 for the treatment of patients aged 60 years or older with CD33-positive relapsed acute AML who were not candidates for conventional chemotherapy (14).

In 2010, the Phase III SWOG S0106 trial revealed that patients treated with Mylotarg showed an increased incidence of hepatic veno-occlusive disease (VOD), with an early mortality rate of 5.7% compared with 1.4% in the control group, and failed to demonstrate a survival benefit compared with chemotherapy alone. As a result, Pfizer Inc. voluntarily withdrew the product from the market in October 2010 (103). In 2017, Mylotarg^®^ was reevaluated and received renewed regulatory approval for the treatment of CD33-positive newly diagnosed AML in adult patients, with the single administration dose reduced from the original 9 mg/m² (used in 2000) to 3 mg/m² (104).

The antibodies used in first-generation ADCs were primarily murine or chimeric, exhibiting significant immunogenicity (15). The linkers, such as the hydrazone bond in Mylotarg, were chemically labile and exhibited poor stability, often leading to premature payload release in the bloodstream and resulting in off-target toxicity (73). The payloads had relatively low potency (e.g., calicheamicin with IC_50_ ≈ 10^-8^ M), contributing to a narrow therapeutic window. Random conjugation techniques resulted in heterogeneous DARs, typically ranging from 0 to 8, yielding highly heterogeneous products (87).

In summary, first-generation ADCs had several limitations, such as an extremely narrow therapeutic window, significant off-target toxicity, strong immunogenicity, and limited efficacy, emphasizing the need for further optimization and development.

### The second-generation ADCs

Second-generation ADCs, represented by brentuximab vedotin and ado-trastuzumab emtansine, were developed through optimizations in mAb design, cytotoxic payloads, and linker technology. These ADCs exhibit improved targeting ability, more potent payloads, and reduced immunogenicity compared with their predecessors.

Brentuximab vedotin selectively binds to the CD30 antigen and is internalized via a clathrin-dependent mechanism (105). It is then transported into endosomes and lysosomes, where the linker is cleaved by cysteine proteases such as cathepsin B. The released free MMAE functions as an ultrapotent antimitotic agent that induces cell-cycle arrest and apoptosis by inhibiting tubulin polymerization.

In the ECHELON-2 trial, which evaluated therapies for CD30-positive peripheral T-cell lymphoma (PTCL), brentuximab vedotin combined with cyclophosphamide, doxorubicin, and prednisone (CHP) was compared with standard CHOP chemotherapy (cyclophosphamide, doxorubicin, vincristine, and prednisone) in CD30-positive PTCL patients. The brentuximab vedotin plus CHP regimen demonstrated a lower risk of death compared with CHOP (106). Based on these results, brentuximab vedotin received approval in 2018 for two additional clinical indications: certain types of peripheral T-cell lymphoma and previously untreated stage III or IV classical Hodgkin lymphoma (cHL) (107).

Second-generation ADCs took advantage of humanized antibodies, substantially reducing immunogenicity. The incorporation of cleavable linkers (e.g., valine-citrulline or VC linkers) enabled tumor-specific payload release with enhanced stability and diminished off-target toxicity. Additionally, more potent payloads improved antitumor efficacy significantly (15).

However, several limitations remained: random conjugation through cysteine or lysine residues continued to produce heterogeneous DAR. This resulted in ADC mixtures containing species with excessively high DAR (associated with increased toxicity) or suboptimal DAR (leading to reduced efficacy), thereby narrowing the therapeutic window. Additionally, some payloads exhibited limited membrane permeability, reducing bystander effects and impairing the elimination of adjacent antigen-negative tumor cells.

.

### The third-generation ADCs

The third-generation ADCs are represented by polatuzumab vedotin, enfortumab vedotin, and fam-trastuzumab deruxtecan. Polatuzumab vedotin targets the CD79b antigen and is conjugated to the microtubule-disrupting agent MMAE via a protease-cleavable dipeptide linker (108). In July 2019, Polivy^®^ (polatuzumab vedotin) received FDA approval for use in combination with bendamustine and rituximab for the treatment of relapsed/refractory diffuse large B-cell lymphoma (DLBCL) in patients who have received at least two prior therapies (109). It was the first ADC approved for DLBCL, the most common type of non-Hodgkin lymphoma (NHL).

Site-specific conjugation enables third-generation ADCs to achieve homogeneous composition, defined DAR (typically 2 or 4), and predictable cytotoxicity (87). These ADCs exhibit reduced off-target toxicity and improved PK profiles for their consistent DAR values. Furthermore, this generation takes advantage of fully humanized antibodies, minimizing immunogenicity compared with earlier chimeric types (15). Meanwhile, more potent payloads have been incorporated, such as PBDs, tubulysins, and novel immunomodulatory agents (52). Certain payloads (e.g., deruxtecan/DXd) possess cell membrane-penetrating capabilities, enabling potent bystander effects that eliminate heterogeneous tumor populations.

However, third-generation ADC technology is characterized by high complexity, significant manufacturing challenges, and extensive production costs. These ADCs also introduce novel toxicity risks. These include unique adverse events such as interstitial lung disease (ILD), which is a concern associated with ultrapotent payloads. A classic example is Enhertu^®^ (fam-trastuzumab deruxtecan-nxki), which carries a black box warning specifically for ILD (110).

## Novel ADCs

### Biepitopic ADCs

**Figure 4:**
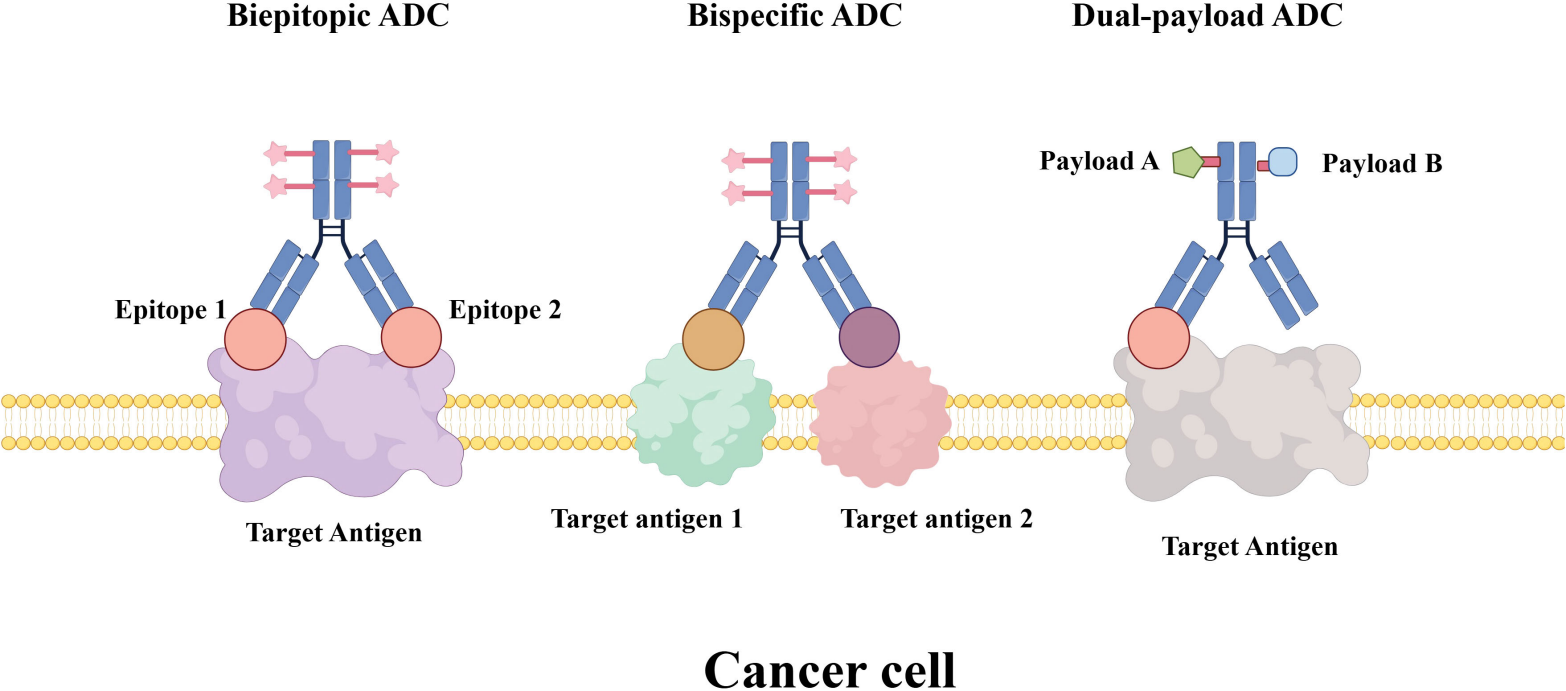
Novel ADC formats for cancer therapy. The illustration compares three advanced ADC design strategies: Biepitopic ADC: an ADC featuring a single antibody engineered to bind two distinct epitopes on the same target antigen, a strategy aimed at enhancing binding avidity and internalization efficiency. Bispecific ADC: an ADC incorporating a bispecific antibody that recognizes two different target antigens, improving tumor selectivity and potential to overcome antigen heterogeneity. Dual-payload ADC: an ADC conjugated with two different cytotoxic payloads (e.g., Payload A and Payload B) via a shared or separate linkers, enabling synergistic mechanisms of action and reduced risk of resistance.

Researchers are exploring novel strategies to overcome resistance to a single-target ADC. Biepitopic ADCs represent an innovative advancement in ADC technology by simultaneously binding to two distinct epitopes on a target antigen (**Figure 4**). This approach is designed to enhance tumor targeting, promote efficient internalization, and overcome drug resistance mechanisms (111). Biepitopic ADCs represent a novel therapeutic strategy in cancer treatment by enabling a single antibody to engage two different epitopes on a tumor-associated antigen. This design enhances binding affinity (particularly in tumors with low antigen expression such as HER2) and improves drug delivery efficiency (112). Although still in early-stage development, preliminary clinical results have shown promising efficacy. For example, TQB2102 has demonstrated a total pathological complete response (tpCR) rate of up to 73.1% in the neoadjuvant treatment of HER2-positive breast cancer (113). Nevertheless, the complex molecular design and manufacturing requirements of biepitopic ADCs present significant challenges. As more research data become available, biepitopic ADCs are expected to provide new therapeutic options for cancer patients.

### Bispecific ADCs

Bispecific antibodies (BsAbs) serve as the carriers for bispecific ADCs (BsADCs). These antibodies possess two distinct antigen-binding domains, enabling them to simultaneously bind to two different antigens and thereby promote the delivery of cytotoxic payloads (114). The simultaneous binding to two different tumor cell surface targets enables bispecific ADCs to achieve greater specificity and more efficient internalization (115). This dual-targeting strategy can promote receptor internalization through cross-linking or enable simultaneous targeting of both tumor cells and components of the tumor microenvironment, leading to synergistic antitumor effects. Even if one target is mutated or downregulated, the other target can still mediate drug delivery. This strategy also helps to overcome resistance (116).

Novel tetravalent BsAbs have been developed to target c-Met/EGFR and c-Met/HER2. These BsAbs combine antibodies that induce rapid internalization and degradation of c-Met with single-chain variable fragments (scFvs) targeting EGFR or HER2 (117, 118). Furthermore, targeting HER2 in combination with other antigens such as B7-H3 and B7-H4 shows promise for broader therapeutic applications (119). For example, an HER2×CD63 bispecific ADC has demonstrated enhanced cytotoxicity in HER2-positive tumor models, underscoring its potential for more precise targeting (120). This HER2×CD63 bispecific ADC is currently under preclinical investigation. SI-B001, a bispecific antibody targeting EGFR and HER3, has been conjugated via a novel acid-cleavable (AC) linker to the topoisomerase I inhibitor ED04, forming the BsADC BL-B01D1 with a DAR of approximately 8. This conjugate exhibits improved targeting precision and safety. SI-B001 has shown promising efficacy in both Phase I and Phase II clinical trials, supporting its advancement to Phase III clinical studies (121).

Bispecific ADCs offer several advantages, including improved tumor selectivity, enhanced internalization efficiency, a potentially wider therapeutic window, and the ability to address tumor heterogeneity and drug resistance. However, the *in vivo* behavior of BsADCs is complex. Although dual targeting may theoretically improve tumor selectivity, it could also introduce novel and unpredictable toxicities, necessitating careful clinical monitoring and management.

### Dual-payload ADCs

Dual-payload ADCs refer to ADCs armed with two distinct payloads that have different mechanisms of action, or the same payload conjugated via different linkers, on a single antibody (122). These ADCs have the potential to function as single therapeutic agents capable of eliciting synergistic effects and overcoming drug resistance in patients with treatment-refractory tumors (123).

For example, one study successfully achieved homogeneous co-conjugation of both MMAE and MMAF to anti-CD30 antibodies, attaining a DAR of 16 (124). These dual-payload ADCs demonstrated potent antitumor activity in a CD30-positive multidrug-resistant naplastic large cell lymphoma mouse xenograft model (125).

Dual-payload ADCs represent a significant advancement beyond conventional ADCs by integrating two distinct classes of payloads, rather than relying solely on cytotoxic agents such as MMAE and MMAF. For instance, a strategic approach co-conjugating a sterlin-like agent with TLR agonists to anti-folate receptor alpha (FolRα) antibodies has shown synergistic antitumor activity and induction of immune memory in mouse models (126).

However, not all dual-payload ADC configurations have demonstrated clear synergistic effects. In some studies combining mechanistically distinct payloads, enhanced efficacy was not observed. Researchers developed an anti-HER2 ADC co-loaded with MMAE and SG3457—an ultrapotent PBD dimer that induces DNA crosslinking damage (127). Similarly, another HER2-targeted ADC was designed to deliver both MMAF and the highly potent topoisomerase II inhibitor PNU-15968. Although both ADCs engaged dual mechanisms of action, neither exhibited superior potency compared with their single-payload counterparts *in vitro* (128).

Multiple challenges must be addressed in the development of dual-payload ADCs. The development of dual-payload ADCs requires complex linker chemistry to ensure both payloads are efficiently released at the correct time and site. Additive or synergistic toxicity from two potent agents may lead to unexpected adverse effects. Furthermore, differences in release kinetics, distribution, metabolism, and clearance profiles between the two payloads complicate PK and pharmacodynamic (PD) relationships, bringing significant challenges for clinical dosing regimen design.

### Immune-stimulating ADCs

**Figure 5:**
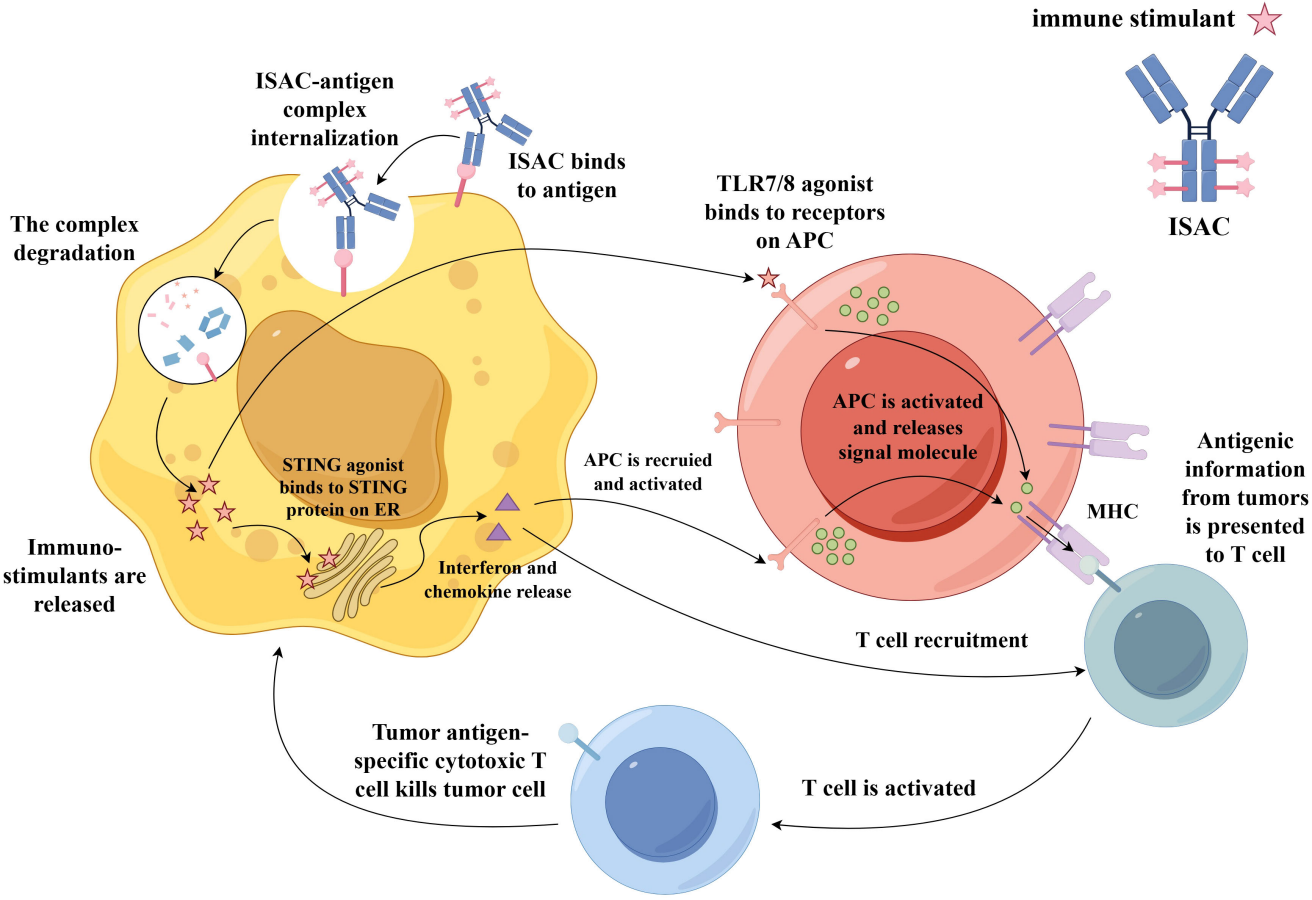
Mechanism of action of an ISAC. After binding specifically to a tumor-associated antigen on the cancer cell surface, the ISAC–antigen complex is internalized. Following degradation of the complex in the lysosome, immuno-stimulatory payloads (such as TLR7/8 or STING agonists) are released. The TLR7/8 binds to receptors on APCs, triggering the recruitment and activation of APCs toward tumor cells. These activated APCs then present tumor-associated antigens via MHC molecules to T cells, activating them into tumor antigen-specific cytotoxic T cell that kills the tumor cell. Additionally, STING agonists bind to the STING protein on the ER, inducing the production of interferons and chemokines. This promotes the recruitment and activation of APCs at the tumor site, which in turn can also activate T cells, further killing tumor cells. Abbreviations: TLR7/8, Toll-like receptor 7/8; APCs, antigen-presenting cells; ER, endoplasmic reticulum.

Immune-stimulating ADCs (ISACs) represent a novel class of therapeutics that share structural similarities with traditional ADCs, comprising three core components: a tumor-targeting antibody, a linker, and an immunostimulatory payload (67). Unlike conventional ADCs that employ cytotoxic agents, ISACs utilize immune agonists or modulators—such as Toll-like receptor (TLR) agonists or STING agonists—as their effector molecules. Upon release from tumor cells into the tumor microenvironment, immunostimulatory payloads are captured by surrounding antigen-presenting cells, leading to their potent activation. Alternatively, these payloads can directly activate innate immune signaling pathways within tumor cells, stimulating the production of chemokines or interferons (129). This response promotes the recruitment and activation of additional immune cells in the tumor microenvironment (**Figure 5**).

Antigen selection is critically important for ISACs and must satisfy criteria including high tumor-specific expression and minimal expression in healthy tissues. IgG1 is the preferred antibody subclass due to its high affinity for Fcγ receptors and extended serum half-life (130). ISACs primarily employ innate immune agonists such as TLR7/8, TLR9, or STING agonists, which effectively promote the priming and recruitment of tumor-specific immune cells. TLR7/8 agonists activate dendritic cells and macrophages, thereby bridging innate and adaptive immunity. TLR9 agonists enhance antigen presentation and cytokine production, whereas STING agonists activate type I interferon pathways to bolster antitumor immunity (131). ISACs are designed to combat tumors through targeted delivery and localized immune activation, theoretically mitigating the systemic toxicities associated with free immune agonists.

Recent preclinical studies have demonstrated that ISACs can effectively promote immune cell infiltration across various tumor models. Despite promising preclinical results, clinical outcomes have been modest. In a Phase 2 trial of BDC-1001 (a HER2-targeted ISAC conjugated to a TLR7/8 agonist), only 1 partial response and 12 cases of stable disease were observed among 57 participants, resulting in an overall response rate of 1.75% (132). Similarly, in a Phase 1 trial of SBT6050 (a HER2-targeted ISAC linked to a TLR8 agonist), among 14 evaluable patients, only one achieved a partial response and three had a stable disease, yielding an overall response rate of 7.1% (133).

The development of ISACs faces significant challenges, such as a narrow therapeutic window. While immune agonists with insufficient potency may be ineffective, excessive activation can lead to severe local or systemic inflammatory reactions, which makes it difficult to identify an optimal balance (131). The relationships among ISAC biodistribution, metabolism, payload release kinetics, and ultimate immunostimulatory effects and toxicity profiles remain highly complex, leading to substantial obstacles for predictive modeling and clinical evaluation (134). Furthermore, ISACs may induce anti-drug antibodies (ADAs), which could compromise efficacy and introduce additional safety concerns (130).

As an emerging strategy in cancer immunotherapy, ISAC development remains at an early stage. Although initial clinical data have fallen short of expectations, ISACs continue to represent a promising direction for novel approaches in tumor immunotherapy.

## Challenges facing ADCs

### Target antigen heterogeneity

Target antigen heterogeneity refers to the significant variation in the expression of target antigens on the surface of tumor cells, which can occur within a single tumor mass or across different metastatic lesions in the same patient (24). In other words, not all tumor cells uniformly express the target antigen for which an ADC is designed. Spatial heterogeneity describes the phenomenon wherein some regions within a tumor exhibit high antigen expression (antigen-positive), whereas other regions show low or no expression (antigen-negative). Temporal heterogeneity refers to the dynamic changes in antigen expression levels over time, which may occur as the disease progresses or in response to treatment (9). ADCs can effectively kill tumor cells with high antigen expression but are often ineffective against those with low or negative expression. These surviving cells may continue to proliferate, ultimately leading to disease relapse. Several strategies have been developed to address target antigen heterogeneity:

1. Utilizing the bystander effect: Employing a membrane-permeable payload allows the cytotoxic agent—once released inside an antigen-positive cell—to diffuse across the cell membrane into the surrounding tumor microenvironment. There, it can enter and kill adjacent antigen-negative tumor cells (77). For example, the DXd payload in trastuzumab deruxtecan (T-DXd/Enhertu^®^) exhibits a potent bystander effect. This property is a key reason for its notable efficacy in HER2-low breast cancer, as it can eliminate tumor cells with heterogeneous HER2 expression (130).

2. Developing payloads with novel mechanisms of action: Arming ADCs with immunostimulatory payloads creates ISACs. Such ADCs target antigen-positive cells to release immune agonists (e.g., TLR or STING agonists), which activate immune cells such as dendritic cells and macrophages in the tumor microenvironment (129, 134). These activated immune cells can attack tumor cells in an antigen-agnostic manner, thereby overcoming heterogeneity.

3. Developing bispecific ADCs: Designing antibodies capable of simultaneously binding two different tumor antigens increases the likelihood of ADC binding even if a tumor cell expresses only one of the target antigens (114, 116). This approach broadens the targetable tumor cell population and reduces the risk of resistance due to loss of a single antigen.

4. Optimizing target antigen: Choosing targets that are more uniformly expressed within tumor tissue can mitigate heterogeneity. Currently, the target antigens of approved ADC drugs are primarily specific proteins overexpressed on typical tumor cells, such as HER2, TROP-2, and nectin-4 in solid tumors, and CD19, CD22, CD33, and CD30 in hematologic malignancies (19, 111). A promising strategy involves targeting mutant proteins uniquely expressed in tumors, as these often exhibit higher levels of ubiquitination and are more readily internalized and degraded compared with their wild-type counterparts. Delivering ADCs specifically to cancer cells expressing such oncogenic mutant proteins may maximize treatment specificity (7, 78). Furthermore, with advances in basic research on tumor immunology, ADC target development has gradually expanded in recent years from classical tumor antigens to include antigens expressed on cells within the tumor microenvironment and cancer stem cells (9, 77).

### Resistance mechanisms

Resistance to ADCs is a major challenge to their clinical efficacy. Gaining a deeper understanding of its mechanisms and developing corresponding strategies are crucial for improving patient outcomes. The primary mechanisms of ADC resistance include the following aspects (1). Target-Related resistance: Due to target antigen downregulation or loss, ADCs cannot recognize and bind to tumor cells. Epitope mutations in the target antigen result in reduced antibody-binding affinity22 (2). Internalization and processing resistance: Decreased internalization efficiency prevents ADC–antigen complexes from entering tumor cells. Lysosomal dysfunction (e.g., impaired acidification) hinders linker cleavage, preventing payload release. Alterations in the expression of lysosomal enzymes (e.g., cathepsins) reduce linker cleavage efficiency87 (3). Payload-related resistance: Upregulation of drug efflux pumps (e.g., P-gp, BCRP) enables cells to pump the payload out, reducing intracellular concentrations77. Mutations in the payload’s target prevent it from exerting its cytotoxic effect. Inactivation of apoptosis pathways or upregulation of antiapoptotic proteins allows cells to evade payload-induced cell death78.

Potential strategies to overcome the aforementioned ADC resistance mechanisms include the development of ADCs targeting novel antigens to expand the target repertoire (e.g., TROP-2, c-Met)117; the utilization of bispecific ADCs capable of simultaneously engaging two tumor antigens to mitigate resistance caused by single-antigen loss (114); and combination therapies with targeted agents, such as proteasome inhibitors, to prevent degradation of the target protein (24). Additional approaches involve the optimization of antibody selection for enhanced internalization efficiency through screening of antibodies with superior cellular uptake capabilities (135); the advancement of novel linker technologies designed for cleavage by a broader spectrum of enzymatic systems; and the substitution of conventional payloads (e.g., microtubule inhibitors like MMAE) with novel agents (e.g., topoisomerase I inhibitors like Dxd) (136). Further strategies include the design of payloads engineered to evade efflux pumps (e.g., P-gp) (137); the exploration of combination regimens with efflux pump inhibitors, although still under clinical investigation due to potential toxicity concerns; and the development of ADCs with potent bystander effects (e.g., DS-8201) to eliminate adjacent antigen-negative tumor cells and address tumor heterogeneity (138).

### Pharmacokinetic complexity

The PK of ADCs represent one of the most complex and challenging aspects of their development. This complexity stems from the fact that ADCs are inherently heterogeneous mixtures that undergo multiple biotransformations *in vivo*. As a result, their PK cannot be adequately described using traditional models developed for conventional mAbs or small-molecule drugs. Following administration, three primary analyte forms can be present in systemic circulation: the intact ADC, the unconjugated (naked) antibody, and the free cytotoxic payload (22).

Intact ADC refers to the complete conjugate structure with the payload attached. It represents the active drug form responsible for target engagement and overall drug exposure. Intact ADCs are primarily eliminated via proteolytic degradation and exhibit a long half-life, typically ranging from days to weeks (87). Unconjugated antibody refers to the bare mAb after the payload has been cleaved and released. It may compete with the ADC for target binding but lacks cytotoxic activity (139). Free payload refers to the small-molecule cytotoxin released into circulation upon linker cleavage. Its PK follows patterns typical of small molecules, with a short half-life (minutes to hours) and very low plasma concentrations that are often challenging to quantify (140). Free payload is primarily metabolized in the liver and eliminated via renal or fecal excretion. This process may be influenced by drug–drug interactions and impaired hepatic or renal function (71).

The release kinetics, distribution, metabolism, and clearance pathways of these three components differ significantly yet are interrelated (141). Together, they shape the overall and safety profile of the ADC. Thus, the PK of ADCs constitute a dynamic, MTI-analyte, and multi-pathway system. A deep understanding of this intricate PK behavior is essential for optimizing therapeutic efficacy and guiding the development of next-generation ADCs.

### Unavoidable side effect

One of the most common adverse effects of ADCs is hematologic toxicity, which manifests as neutropenia (the most frequent), anemia, and thrombocytopenia. These effects are likely due to the released payload—often an antimitotic agent—affecting rapidly dividing bone marrow hematopoietic cells (142). Regular monitoring of complete blood counts is essential during treatment. Granulocyte colony-stimulating factor (G-CSF) may be used for the prevention or treatment of neutropenia. Dose delays or adjustments may be necessary based on clinical indications (53).

Ocular toxicity is linked to specific payloads (e.g., MMAE, MMAF) and may include dry eye syndrome, keratopathy, and blurred vision (72). This may result from payload distribution to ocular tissues via tear secretion or systemic circulation, affecting proliferating corneal epithelial cells. Prophylactic use of artificial tears is recommended. In severe cases, ophthalmologic consultation is necessary, and ADC therapy may need to be interrupted or discontinued (143).

Hepatotoxicity, which is related to the hepatic metabolism of the payload or Fc-mediated uptake of the ADC (144), may manifest as elevated transaminases and bilirubin levels. Therefore, liver function should be monitored regularly during treatment, with dose adjustments or treatment interruptions implemented based on severity. Gastrointestinal adverse reactions are also common, including nausea, vomiting, diarrhea, and decreased appetite (53). Patients should be managed with prophylactic antiemetics and active supportive measures, including antidiarrheal agents and fluid replacement.

ILD/pneumonitis is one of the most serious and potentially life-threatening adverse reactions and requires heightened vigilance. It is strongly associated with certain ADCs, as highlighted by the boxed warning for Enhertu (T-DXd)-related ILD (110). Payloads with high membrane permeability (such as DXd and SN-38) can be released from target cells and diffuse into surrounding normal lung tissue, causing DNA damage and cell death, which leads to intense inflammatory and fibrotic responses. This is the primary supposed mechanism for Enhertu (T-DXd)-related ILD. Microtubule inhibitors (such as MMAE and DM1) may also result in lung injury by injuring normal alveolar epithelial cell function or inducing vascular leakage (145). Early recognition is critical. Upon symptom onset, treatment should be interrupted immediately, followed by radiographic evaluation and corticosteroid therapy (146).

Dermatologic toxicity manifests associated with ADCs present as rash, pruritus, and dryness; in severe cases, it may lead to extensive skin detachment and mucosal erosion (e.g., enfortumab vedotin) (53). This may be related to the expression of Nectin-4 in skin keratinocytes, non-specific killing caused by the ADC “bystander effect”, and synergistic exacerbation of inflammation due to immune activation (147). Before treatment, it is essential to assess the patient’s baseline skin condition and risk factors. During treatment, regular skin examinations should be conducted, with particular attention to new rashes or changes in existing rashes (148).

Peripheral neuropathy presents as tingling or numbness in the fingers or toes, as well as muscle weakness (149). This may be associated with MMAE payload disrupting microtubule function, affecting neuronal axonal transport and nerve fiber integrity (150). Before treatment, it is necessary to evaluate the patient’s existing neuropathy symptoms and risk factors. During each follow-up, actively inquire whether the patient has symptoms such as numbness, tingling, burning pain, or weakness in the hands and feet, and perform simple neurological examinations (e.g., pinprick sensation, vibration sense, tendon reflexes) (148).

Although not all ADCs induce hyperglycemia, those ADCs incorporating MMAE carry a well-established risk. MMAE, an antimicrotubule agent that inhibits cell division, can disrupt microtubule function. Pancreatic beta cells, which secrete insulin, are notably sensitive to such disruption despite their low rate of division (151). Patients with preexisting risk factors, such as a history of diabetes, obesity, or insulin resistance, are generally at increased risk for hyperglycemic events. ADC therapy may exacerbate these underlying metabolic disorders (53). Before the treatment with such ADCs, patients should receive a baseline assessment that includes blood glucose and glycated hemoglobin measurements, in addition to an evaluation of their diabetes history and risk profile. During therapy, blood glucose levels should be monitored regularly, particularly proximate to each treatment cycle (148).

## Combination therapy of ADCs with immune checkpoint inhibitors

With the precise delivery of potent cytotoxic drugs into tumor cells, ADCs have revolutionized cancer treatment greatly. However, the complexity and adaptability of tumors often lead to the failure of single-agent therapies (152). Therefore, combining ADCs with other therapies—such as ICIs that enhance immune response, targeted drugs that block different signaling pathways, or traditional chemotherapy—has become an essential strategy to overcome drug resistance and enhance the depth and persistence of response (153).

ICIs have a completely different mechanism from that of ADCs. ICIs are designed to enhance the inherent antitumor activity of the immune system by removing inhibitory signals of T-cell activation to restore cytotoxic immune effector function against cancer cells. ADCs can reduce immunosuppressive cells, increase CD8+ T-cell infiltration, enhance the response of tumors to immunotherapy, and remodel the tumor microenvironment, thereby enhancing the efficacy of ICIs (154).

PD-1/PD-L1 inhibitors are a type of immune checkpoint inhibitor that can block the interaction between PD-1 and PD-L1 to prevent the immune escape of tumor cells. The combination therapy of ADCs with PD-1/PD-L1 inhibitors represents a cutting-edge direction in current cancer immunotherapy, demonstrating significantly enhanced antitumor efficacy through synergistic mechanisms (155). The combination regimen of enfortumab vedotin (EV), an ADC directed to Nectin-4, and pembrolizumab, a PD-1 inhibitor, was evaluated in EV-302, a Phase 3 study in patients with locally advanced or metastatic urothelial cancer. The combination showed a statistically significant and clinically meaningful improvement in overall survival (OS), progression-free survival (PFS), and the key secondary endpoint of objective response rate (ORR) compared with chemotherapy. The ORR was higher in the combination group than in the chemotherapy group (68% vs. 44%). The median PFS was 12.5 months in the combination group versus 6.3 months in the chemotherapy group (HR = 0.45), whereas the median OS was 31.5 versus 16.1 months (HR = 0.47) (156).

Sacituzumab govitecan, a Trop-2-directed ADC with a topoisomerase I inhibitor payload, improves PFS and OS compared with chemotherapy in patients with pretreated metastatic triple-negative breast cancer (TNBC) (157). The open-label, international, multicenter, randomized Phase III trial (AFT-65/ASCENT-05/OptimICE-RD) will determine whether the combination of sacituzumab govitecan and pembrolizumab (a PD-1 inhibitor) can improve interval disease-free survival compared with pembrolizumab alone or in combination with capecitabine in patients with stage II-III TNBC who have residual invasive disease after neoadjuvant therapy. This clinical trial is currently underway (158). In a study of patients treated with RC-48-ADC and toripalimab, HER2 was positive in 59% of patients, and the objective response rate was 73.2% (159). These results demonstrate promising efficacy for this combination regimen. Other combination trials are ongoing, and their results are eagerly awaited.

The combination of enfortumab vedotin (EV) and pembrolizumab brings new hope to patients with advanced urothelial carcinoma. However, its associated safety issues—particularly adverse events such as cutaneous toxicity (147), neuropathy (150), and hyperglycemia (151)—require closer attention and proper management by clinicians, as previously discussed in the section on unavoidable side effects of ADC-based therapeutics.

Overall, the combination of ADCs and ICIs is a highly promising direction in the field of cancer therapy. Its core advantage lies in a strong synergistic effect at the mechanism level, which has been proven in multiple clinical trials of specific tumor types to significantly improve efficacy. Despite challenges in safety, biomarker development, and protocol optimization, this strategy shows great promise for improving cancer therapy and becomes a key direction for future research and clinical practice.

## Conclusion

ADCs represent a pioneering class of targeted anticancer therapeutics. ADCs have been designed to combine the specificity of monoclonal antibodies with the potent cytotoxicity of chemotherapeutic agents. With the selective delivery of highly toxic payloads to tumor cells expressing specific antigens, ADCs significantly improve the therapeutic index and minimize damage to healthy tissues. ADCs have achieved remarkable clinical and commercial success and significantly influenced the pharmaceutical industry. However, ADCs still face numerous challenges. Target antigen heterogeneity can limit efficacy and drive resistance. Mechanisms of resistance include antigen downregulation, impaired internalization, payload efflux, and altered apoptosis pathways. ADC pharmacokinetics are complex due to the coexistence of intact conjugates, naked antibodies, and free payloads, each with distinct behaviors. Toxicity is a major concern. Common adverse effects include hematologic toxicity, neuropathy, ocular damage, hepatotoxicity, and pneumonitis.

To overcome these limitations, innovative strategies are being explored. The conjugation technology field is rapidly moving from random conjugation toward site-specific strategies to produce homogeneous ADCs with excellent stability and PK. New ADCs are under exploration, such as bsADCs, dual-payload ADCs, and ISACs. The combination of ADCs with ICIs such as PD-1/PD-L1 inhibitors has demonstrated synergistic efficacy by enhancing antitumor immune responses. Trials of enfortumab vedotin plus pembrolizumab have shown significantly improved response and survival rates in urothelial carcinoma and other cancers. In conclusion, ADCs have ushered in a new era of targeted cancer therapy, offering substantial benefits over traditional treatments. Ongoing research focuses on optimizing antibody engineering, linker stability, payload potency, and conjugation methods to enhance efficacy and reduce toxicity. The integration of ADCs with other therapeutic modalities holds great potential to address resistance, improve outcomes, and expand treatment options for cancer patients.
